# Sphingosine Kinase 2 and Ceramide Transport as Key Targets of the Natural Flavonoid Luteolin to Induce Apoptosis in Colon Cancer Cells

**DOI:** 10.1371/journal.pone.0143384

**Published:** 2015-11-18

**Authors:** Loubna Abdel Hadi, Clara Di Vito, Giovanni Marfia, Anita Ferraretto, Cristina Tringali, Paola Viani, Laura Riboni

**Affiliations:** 1 Department of Medical Biotechnology and Translational Medicine, LITA-Segrate, University of Milan, Milan, Italy; 2 Laboratory of Experimental Neurosurgery and Cell Therapy, Neurosurgery Unit, Fondazione IRCCS Cà Granda, Ospedale Maggiore Policlinico Milan, University of Milan, Milan, Italy; 3 Department of Biomedical Sciences for Health, LITA-Segrate, University of Milan, Milan, Italy; Medical University of South Carolina, UNITED STATES

## Abstract

The plant flavonoid luteolin exhibits different biological effects, including anticancer properties. Little is known on the molecular mechanisms underlying its actions in colorectal cancer (CRC). Here we investigated the effects of luteolin on colon cancer cells, focusing on the balance between ceramide and sphingosine-1-phosphate (S1P), two sphingoid mediators with opposite roles on cell fate. Using cultured cells, we found that physiological concentrations of luteolin induce the elevation of ceramide, followed by apoptotic death of colon cancer cells, but not of differentiated enterocytes. Pulse studies revealed that luteolin inhibits ceramide anabolism to complex sphingolipids. Further experiments led us to demonstrate that luteolin induces an alteration of the endoplasmic reticulum (ER)-Golgi flow of ceramide, pivotal to its metabolic processing to complex sphingolipids. We report that luteolin exerts its action by inhibiting both Akt activation, and sphingosine kinase (SphK) 2, with the consequent reduction of S1P, an Akt stimulator. S1P administration protected colon cancer cells from luteolin-induced apoptosis, most likely by an intracellular, receptor-independent mechanism. Overall this study reveals for the first time that the dietary flavonoid luteolin exerts toxic effects on colon cancer cells by inhibiting both S1P biosynthesis and ceramide traffic, suggesting its dietary introduction/supplementation as a potential strategy to improve existing treatments in CRC.

## Introduction

CRC is one of the most common neoplasia and a leading cause of death worldwide. This cancer was recognized as, and still remains, an environmental cancer, its incidence being increased parallel to economic development, with the majority of cases occurring in industrialized countries, and mainly attributable to the diet [1, 2]. Numerous studies have linked abundant consumption of foods from plant origins with decreased risk of developing various cancers, a chemo-preventive effect that is related to the high content of several phytochemicals with potent anticancer properties [3], including compounds of the flavonoid family [4, 5]. One of the most common component of this family is luteolin (3′,4′,5,7-tetrahydroxyflavone), which is present at high levels in common fruits, vegetables and herbs, and exhibits a wide spectrum of effects, including anticancer activities [6, 7]. Luteolin anti-carcinogenic properties expand over a wide range of malignancies and are associated to multiple effects, such as inhibition of cell proliferation, angiogenesis, metastasis, induction of apoptosis, and sensitization to chemotherapy [6, 7]. Notwithstanding, the molecular mechanisms underlying luteolin actions, and particularly those related to its chemotherapeutic potential, remain largely unclear.

In different tumor cells, ceramide, the key intermediate of sphingolipid metabolism, has been shown to act as cellular mediator of multiple anticancer compounds, being able to regulate different signaling pathways, and leading to cell cycle arrest and apoptosis [8, 9]. Several enzymes in different subcellular locations are involved in the control of ceramide level [10]. The pro-apoptotic and tumor-suppressing effects of ceramide are antagonized by S1P, a pro-mitogenic and survival factor for a variety of cell types [11–13]. S1P metabolism is directly linked to that of ceramide, its biosynthesis requiring sphingosine, derived from ceramide hydrolysis, and SphKs (isoform SphK1 or SphK2). S1P exhibits both intracellular and extracellular actions, primarily through activation of pro-mitogenic and pro-survival signaling [11, 14]. The proper regulation of the sphingolipid rheostat, that is the balance between S1P and ceramide, is essential for cellular homeostasis, and plays a fundamental role in regulating cell properties and fate [11, 13].

Ceramide levels have been reported to be significantly reduced in CRC when compared with normal colon tissue [15], and several chemotherapeutics were found to impact on ceramide metabolism and promote its accumulation in colon cancer cells (reviewed in [16]). Moreover, S1P stimulates growth, invasion and survival of colonic tumor cells [17, 18], and SphK1 and S1P lyase are up- and down-regulated, leading to S1P accumulation in CRC [19, 20]. These pieces of evidence suggest that the unbalance of the sphingolipid rheostat favor CRC.

In spite luteolin appears promising as chemotherapeutic in some cancer cells [7], little is known on the role of the sphingolipid rheostat on its actions, and particularly in CRC. The present study was designed to investigate the potential role of both ceramide and S1P in luteolin cytotoxicity in CRC. Using human Caco-2 cells as CRC model, our study reveals for the first time the sphingolipid rheostat as a target of luteolin cytotoxic effects.

## Materials and Methods

### Materials

All reagents were of highest available analytical grade. Eagle’s Minimum Essential Medium (EMEM), brefeldin A (BFA), free fatty acid-BSA (FFA-BSA), N-acetyl-D-erythro-sphingosine (C2-Cer), N-hexanoyl-D-erythro-sphingosine (C6-Cer), O-tricyclo[5.2.1.02,6]dec-9-yl dithiocarbonate potassium salt (D609), Hoechst 33342, luteolin, pertussis toxin (PTX), and common chemicals were from Sigma Aldrich (St. Louis, MO, USA). S1P was purchased from Enzo Life Sciences (Farmingdale, NY, USA), and caged S1P from Alexis Biochemicals (Plymouth Meeting, PA, USA). High performance TLC (HPTLC) silica gel plates and all solvents were from Merck (Darmstadt, Germany). Fetal calf serum (FCS) was from EuroClone (Milan, Italy). LY294002, SEW2871, and W123 were from Cayman Chemical (Ann Arbor, MI, USA), and N-(4,4-difluoro-5,7-dimethyl-4-bora-3a,4a-diaza-*s*-indacene-3-pentanoyl)-sphingosine (BODIPY-C_5_-Cer) from Life Technologies (Monza, Italy). ^3^H-serine (30 Ci/mmol), ^3^H-D-erythro-sphingosine (^3^H-Sph) (20.0 Ci/mmol) and D-erythro-[4,5-^3^H]dihydrosphingosine (60.0 Ci/mmol) were from PerkinElmer Life Sciences (Boston, MA, USA) and American Radiolabeled Chemicals, Inc. (St. Louis, MO, USA).

### Cell culture

The human colon carcinoma cell line Caco-2 (BS TCL 87) was obtained from the Istituto Zooprofilattico Sperimentale della Lombardia e dell'Emilia Romagna (Brescia, Italy), and maintained in EMEM supplemented with 15% FCS, 2 mM L-glutamine, 1 mM sodium pyruvate, 100 μg/mL streptomycin, 100 U/mL penicillin, and 0.25 μg/mL amphotericin-B at 37°C in a humidified atmosphere of 5% CO_2_. Caco-2 cells were plated at 1.25 × 10^4^/cm^2^, and used as either colon cancer cell model (CC cells) (at confluence, 3 days after plating), or differentiated enterocytes (DEs) (21 days after confluence) [21]. Intestinal differentiation was confirmed by evaluating specific morphological features and biochemical markers as previously reported [21].

### Cell treatments

Stock solutions of the administered molecules were prepared by dissolving them as follows: luteolin, SEW2871, W123, and caged S1P in DMSO, C2-Cer and BFA in absolute ethanol, C6-Cer as 1:1 complex with FFA-BSA, and S1P in FFA-BSA (4 mg/ml in PBS). At the time of the experiments, stock solutions were diluted in fresh medium to the appropriate concentration and administered to cells. In parallel experiments, cells were incubated with diluted vehicles as control. All solvents, at the used final concentrations, were found without effect on either sphingolipid metabolism or cell survival. When used, W123 and PTX were administered 1 h before and during treatments. For intracellular S1P generation, CC cells were loaded with caged S1P (in EMEM containing 1 mg/ml FFA-BSA) at 37°C for 2 h. The medium was then removed, and photolysis was performed by a 30-s UV pulse at 360 nm with the maximal intensity.

### Determination of cell viability and apoptosis

Cell viability was determined by the MTT assay. Briefly, after treatment for indicated period of time, the medium was replaced by MTT dissolved in fresh medium (0.8 mg/ml) for 4 hours. The formazan crystals were then solubilized in isopropanol/formic acid (95:5 v/v) for 10 minutes and the absorbance (570 nm) was measured using a microplate reader (Wallack Multilabel Counter, Perkin Elmer, Boston, MA, USA).

To evaluate apoptosis, Hoechst staining and analysis of caspase 3 cleavage were performed. In particular, cells grown on glass coverslips were labeled with 10 μM Hoechst 33342 dye for 15 min at 37°C, in the dark. After washing with PBS, cells were fixed with 0.5% glutaraldehyde in PBS (10 min, 4°C). The nuclear morphology was examined by a fluorescence microscope (Olympus BX-50), equipped with a fast high-resolution CCD camera (Colorview 12) and an image analytical software (Analysis from Soft Imaging System GmbH). Caspase-3 cleavage was evaluated by western blotting (see below).

### Ceramide quantification and metabolic studies

In order to evaluate ceramide content and sphingolipid metabolism, cell sphingolipids were labeled with 25 nM ^3^H-Sph (0.5 μCi /ml) or 200 nM L-^3^H-serine (6.0 μCi/ml) as previously reported [22, 23], for different times. In particular, for ceramide quantification, we performed a 6 h pulse followed by a 24 h chase, a condition warranting a steady-state metabolic labeling. For metabolic studies, pulse experiments were performed for 1–2 h. At the end of pulse/chase time, the medium was carefully collected, cells were scraped off the plate and sphingolipids and S1P were extracted and partially purified as previously described [22, 24]. After counting for radioactivity by liquid scintillation, the final organic and aqueous phases were submitted to HPTLC, using chloroform/methanol/water 55:20:3 (by vol.) and in n-butanol/acetic acid/water 3:1:1 (by vol.) for the separation of complex sphingolipids and SlP, respectively. HPTLC plates were then submitted to digital autoradiography with Beta-Imager 2000 instrument (Biospace, Paris, FR). The radioactivity associated with individual lipids was determined with the M3-Vision software provided with the instrument. ^3^H-sphingolipids were identified by co-migration with internal standards chromatographed in the same plate. Ceramide content was determined by calculating the steady-state ^3^H-ceramide/^3^H-sphingomyelin ratio, and multiplying it for endogenous sphingomyelin content (22, 23). Similar ceramide levels (different for less than 12%) were obtained after cell labeling at equilibrium with ^3^H-Sph and ^3^H-serine.

^3^H-S1P degradation was evaluated as tritiated water by fractional distillation of the aqueous phase from the culture medium, and measuring the radioactivity by liquid scintillation [22]. Control experiments with added ^3^H_2_O showed that no loss of tritium by evaporation occurred under the used experimental conditions.

### Intracellular localization of fluorescent ceramide

The ER-Golgi transport of ceramide was qualitatively evaluated with BODIPY-C_5_-Cer as previously reported [25]. Briefly, cells grown on glass coverslips were loaded with 2.5 μM BODIPY-C_5_-Cer for 30 min, at 4°C, and then incubated in conditioned medium (30 min at 37°C), in the absence or presence of luteolin. The specimens were then fixed with glutaraldehyde, and analyzed by fluorescence microscopy (see above).

### SphK activity

Cells were harvested in SphK buffer (20 mM Tris-HCl, pH 7.4 containing 40 mM β-glycerophosphate, 1 mM EDTA, 0.5 mM deoxypyridoxine, 15 mM NaF, 1 mM β-mercaptoethanol, 1 mM Na_3_VO_4_, 0.4 mM PMSF, 10% glycerol, and complete protease inhibitors), and disrupted by freeze-thawing. Equal amounts of proteins [26] were assayed for SphK activity, using experimental conditions known to selectively favor SphK1 or SphK2 activity [27, 28]. The mixture was incubated at 37°C for 15–30 min. The reaction was terminated by the addition of chloroform/methanol (2:1, by vol.), followed by double partitioning, first in alkaline and then in acidic conditions. S1P in the final organic phase was resolved by HPTLC, and quantified by digital autoradiography. Background values were determined in negative controls in which ATP was not added to the reaction mixture.

### Western blotting

Cells were lysed (20 min at 4°C) in Tris-buffered saline (20 mM Tris-HCl, pH 7.4, 150 mM NaCl) containing, 1% Nonidet P-40, 10 mM NaF, 1 mM Na_3_VO_4_, 10 mM sodium pyrophosphate, 1 mM PMSF, and protease inhibitors. After centrifugation, supernatants were assayed for proteins [26], and equal amount of proteins (15–30 μg) were submitted to SDS-PAGE on 10% polyacrylamide gel. After transfer onto nitrocellulose membranes, samples were incubated (overnight, 4°C) with the following antibodies: anti-SphK1 and anti-SphK2 (Abcam, Cambridge, UK), anti-phospho-Akt, anti-caspase-3 antibody (Cell Signaling Technology, Inc., Danvers, MA), and then with an anti-HRP-conjugated antibody (Santa Cruz Biotechnology, Santa Cruz, CA). Anti-β-actin (Sigma Aldrich, St. Louis, MO) and anti-GAPDH antibodies (Santa Cruz Biotechnology, Santa Cruz, CA) were used as loading control. Immunoreactive signals were visualized by Supersignal West Femto (Thermo Scientific (Rockford, IL), and exposure to Kodak Biomax film (Rochester, NY). Immunoreactive band density was determined with a densitometer (Bio-Rad Laboratories, Hercules, CA; Quantity One software).

### Statistical analysis

Data are expressed as mean ± SD of at least three independent experiments in duplicate. Data were analyzed using StatMate software, version 4.0 (GraphPad). Statistical analysis was carried out using the Student’s *t*-test. Differences were considered statistically significant at *p* < 0.05.

## Results

### Luteolin induces apoptosis in CC cells

**Fig 1:**
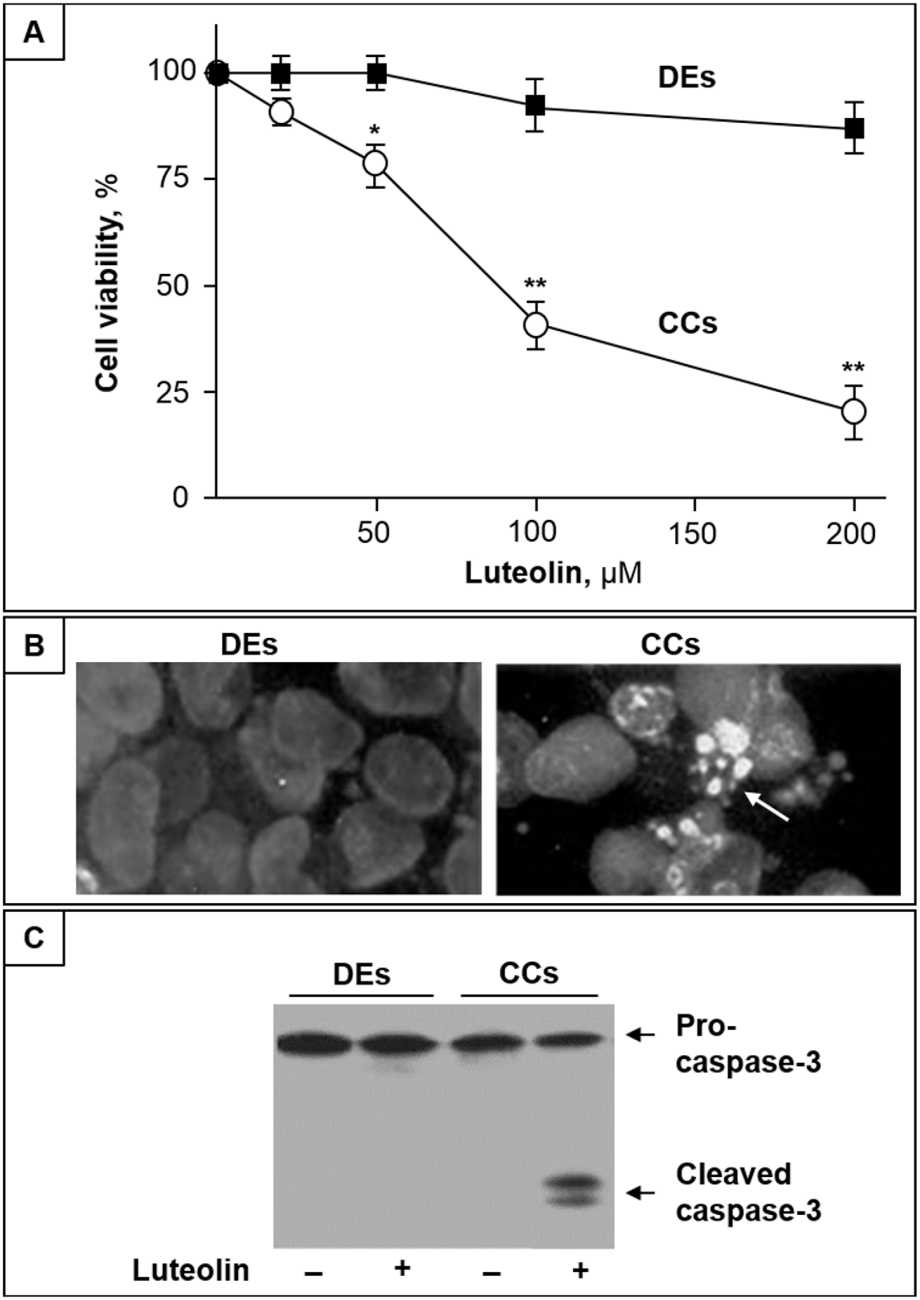
Luteolin induces apoptosis in CC cells but not in DEs. (A) CC cells (CCs) and DEs were treated with different concentrations of luteolin, and after 48 h cell viability was assayed by MTT. Data are the mean ± SD of three independent experiments; *, p < 0.05 and **, p < 0.01 vs. control. (B) Representative microscopic images of DEs and CCs stained with Hoechst 33342 after treatment with 100 μM luteolin for 36 h. (C) Western blot analysis of Pro-caspase-3 and active Caspase-3 intracellular levels of DEs and CCs treated or not with 100 μM luteolin for 36 h.

We first evaluated the effect of luteolin on DEs and CC cell viability. As shown in Fig 1A, up to 100 μM luteolin exerts no evident toxic effect in DEs, and even at the highest concentration (200μM), a modest, if any, cytotoxic effect was observed. To the opposite, in the same of concentration range, luteolin induced a dose-dependent decrease of CC cell viability (Fig 1A). At luteolin concentrations higher than 20 μM, changes in CC cell morphology characteristic of apoptotic cells, including shrinkage and extensive detachment from the culture substratum, were observed (not shown). After 24 h treatment with cytotoxic doses of luteolin, fluorescence microscopic analyses with Hoechst 33342 revealed that CC cells presented apoptotic morphological changes, with condensation and fragmentation of nuclei, and exhibited brilliant blue fluorescence (Fig 1B, right). Conversely, in the same conditions, DEs were found with normal appearance, and the fluorescent dye stained morphologically normal nuclei, with a dimly blue fluorescence (Fig 1B, left). In addition, immunostaining of caspase-3 revealed that luteolin induced pro-caspase-3 activation in CC cells, but not in DEs (Fig 1C).

### Accumulation of ceramide is involved in luteolin-induced apoptosis

**Fig 2:**
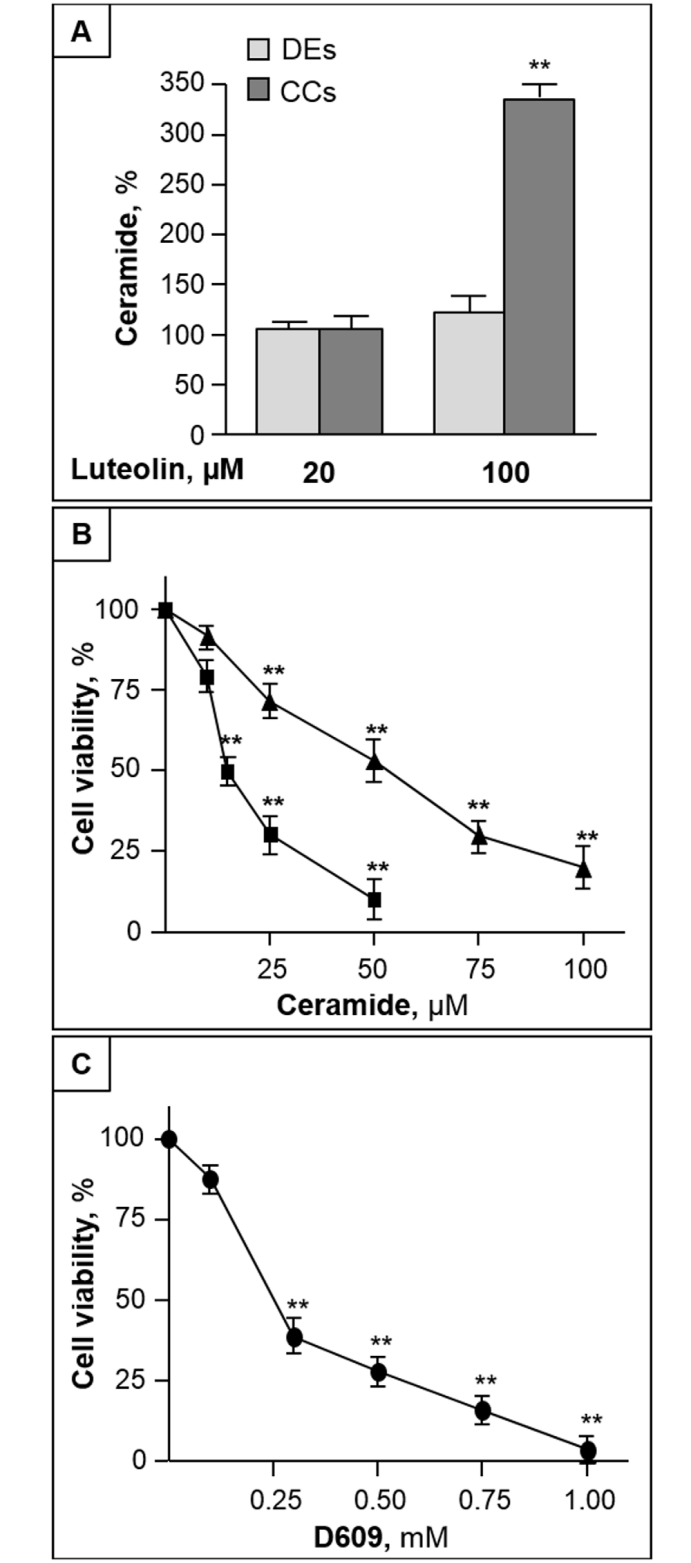
Luteolin increases ceramide level in CC cells, and induced elevation of ceramide leads to CC cell toxicity. (A) DEs and CC cells were treated with luteolin, and, after 24 h, cellular ceramide was quantified. (B) and (C) CC cells were treated with different concentrations of C2-Cer (B, square), C6-Cer (B, triangle) or D609 (C), and after 48 h cell viability was assessed by MTT. All data are the mean ± S.D. of three independent experiments. *, p < 0.05; **, p < 0.01 vs control.

In the used experimental conditions, the basal level of ceramide was significantly lower in CC cells than in DEs (2.45±0.38 and 4.73±0.59 nmol/mg protein, respectively). After 24 h treatment with cytotoxic doses of luteolin, we found that the ceramide level of CC cells was significantly increased (more than 3-fold) by luteolin at toxic, but not sub-toxic doses (Fig 2A). The luteolin-induced ceramide increase was measurable in CC cells prior to evident morphological and nuclear changes. In the same conditions of luteolin treatment, no significant variation in ceramide content was observed in DEs (Fig 2A). These results prompted us to investigate the mechanisms underlying luteolin-induced ceramide increase, focusing on CC cells.

The exposure of CC cells to increasing doses of cell permeable C2- and C6-Cer resulted in a dose-dependent cytotoxic effect (Fig 2B). The potency of these ceramide analogues to induce CC cell death was inversely related to their length of their acyl chain, as expected on the basis of their cell permeability. In addition, cell treatment with D609, an inhibitor of sphingomyelin synthase [29], induced a cytotoxic effect too (Fig 2C). D609 treatment (0.5 mM for 24 h), induced a significant increase (1.94-fold, p < 0.01) of ceramide (from 2.45±0.38 to 4.76±0.59), indicating that this treatment was effective in elevating intracellular ceramide. CC cells treated with ceramides or D609 showed chromatin condensation and caspase-3 activation (not shown), indicating an induced increase of cellular ceramide was able to mimic luteolin in inducing apoptosis.

### Luteolin inhibits ceramide metabolism to complex sphingolipids

**Fig 3:**
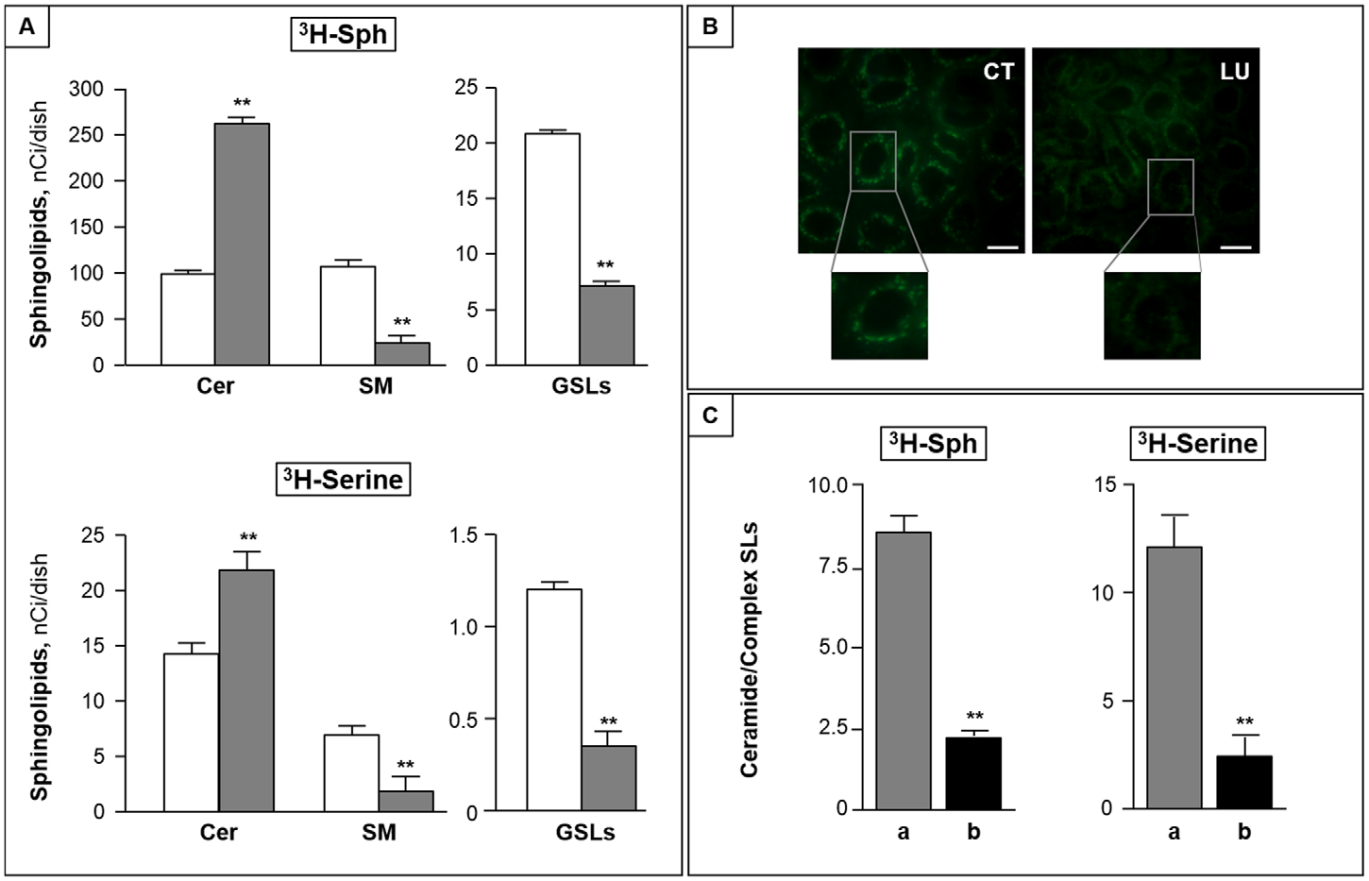
Luteolin inhibits ceramide metabolism to complex sphingolipids. (A) CC cells were pulsed with 25 nM ^3^H-Sph (upper panel) or 200 nM ^3^H-serine (lower panel) in the absence (white bar) or presence (grey bar) of 100 μM luteolin for 2 h. At the end the content of cellular radiolabeled ceramide (Cer), sphingomyelin (SM) and glycosphingolipids (GSLs) was measured. **, p < 0.01. (B) CC cells were incubated in the absence (CT) or presence of luteolin (LU), then with BODIPY-C_5_-Cer and analyzed by fluorescence microscopy (bar, 10 μm). (C) CC cells were incubated without (a) or with (b) BFA (1 μg/ml) for 30 min, and then pulsed with ^3^H-Sph or ^3^H-serine with luteolin (100 μM) for 2 h. The ceramide/complex sphingolipid ratio is reported. Data are the mean ± S.D. of two independent experiments. **, p < 0.01.

To investigate the mechanism underlying the ceramide accumulation induced by luteolin, we then performed pulse experiments with labeled sphingosine. We found that ^3^H-Sph was rapidly incorporated into CC cells, independently of luteolin treatment. Indeed, total incorporated radioactivity accounted for 373 ± 20 and 385 ± 25 nCi/dish in control and luteolin-treated CC cells, respectively. In both cases, after one hour pulse, the level of intracellular ^3^H-Sph represented less than 5% of total incorporated radioactivity (not shown), indicating an efficient sphingosine metabolism occurred in CC cells, and was unaffected by luteolin. The bulk of incorporated radioactivity was associated to N-acylated sphingosine derivatives, mainly represented by ceramide and sphingomyelin, and, in much lower amounts, by glycosphingolipids. Luteolin treatment significantly increased the amount of radioactivity incorporated into N-acylated metabolites (213.7 ± 17.3 and 282.4 ± 24.9 nCi/dish, in control and luteolin-treated cells), and modified its distribution among different sphingosine metabolites. Indeed, in luteolin-treated cells, radiolabeled ceramide was found more than 2-fold higher than that of control cells (Fig 3A, upper panel), and was paralleled by a significant reduction of complex sphingolipids, including both sphingomyelin and glycosphingolipids (Fig 3A, upper panel). Similar results were obtained in pulse experiments with labeled serine, used as precursor of the de novo sphingolipid synthesis (Fig 3A, lower panel). On the whole, these variations resulted in a significant increase of the ceramide/complex sphingolipid ratio in luteolin-treated cells compared to control ones (more than 10- and 7-fold after ^3^H-Sph and ^3^H-serine pulse, respectively, p < 0.001).

After a 2 h pulse with L-^3^H-serine, control and luteolin-treated cells incorporated similar amounts of radioactivity into total sphingolipids (22.3±2.7 and 24.9±3.0 nCi/dish). In the used experimental conditions, ^3^H-ceramide represented the major labeled sphingolipid, and was found significantly increased in luteolin-treated cells (Fig 3A, lower panel). As in the case of ^3^H-Sph pulse, ceramide elevation was accompanied by a significant reduction of both sphingomyelin and glycosphingolipids (Fig 3A, lower panel).

### Luteolin impairs the ER-Golgi traffic of ceramide

On the bases of the above results, it was of interest to investigate whether the decrease of ceramide metabolism to complex sphingolipids is due to alterations of its transport from the ER to the Golgi apparatus. To this purpose, we first studied the effect of luteolin on the intracellular transport of BODIPY-C_5_-Cer, a fluorescent ceramide analogue able to mimic the ER-Golgi traffic of natural ceramide in living cells [30]. After labeling control cells with BODIPY-C_5_-Cer, highly fluorescent intracellular vesicles accumulated in compact perinuclear structures, representative of the Golgi apparatus (Fig 3B, left), indicating the normal exit of ceramide from the ER. By contrast, in luteolin-treated cells a fluorescence spreading throughout the cells with a diffuse reticular pattern, and accompanied by a reduction of perinuclear fluorescence was evident (Fig 3B, right). These variations, together with the results of the pulse study, are consistent with the hypothesis that cytotoxic doses of luteolin impair the ceramide transport from the ER to the Golgi. To further substantiate this, we investigated the effect of BFA, which disrupts the Golgi by inducing its fusion with the ER [31], on luteolin-induced impairment of ceramide metabolism. We found that BFA significantly reduced luteolin-induced ceramide accumulation, and this was paralleled by the elevation of both sphingomyelin and glucosylceramide. As a consequence, in the presence of BFA, the luteolin-induced elevation of the ceramide/complex sphingolipids ratio was markedly reduced (Fig 3C).

### Akt is involved in luteolin-induced impairment of ceramide traffic

**Fig 4:**
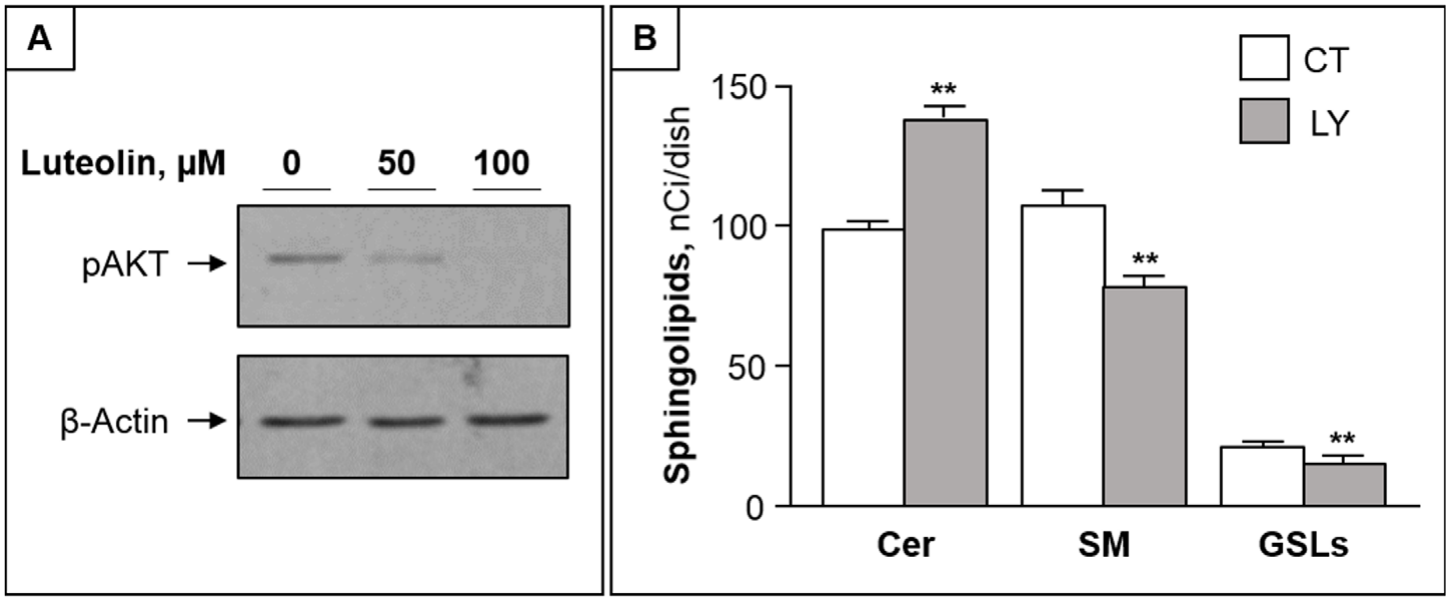
Akt inhibition by luteolin is involved in its effect on ceramide metabolism. (A) CCs were treated with 50 and 100 μM of luteolin for 2 h and submitted to immunoblotting with anti-pAKT antibodies. β-actin was used as loading control. (B) Cells were submitted to pulse with ^3^H-Sph in the absence (CT) or presence of LY294002 for 2h. The levels of ceramide (Cer), sphingomyelin (SM) and glycosphingolipids (GSLs) are reported as mean ± S.D. of at least three independent experiments. **, p < 0.01 vs CT cells.

As the activation of the serine-threonine kinase Akt (protein kinase B) is crucially involved in survival signaling in various cell types [32], we next investigated whether Akt is involved in luteolin toxicity. We found that luteolin treatment caused a dose-dependent reduction of phosphorylated Akt in CC cells (Fig 4A). Furthermore, LY294002, a specific inhibitor of PI3K/Akt, was able to mimic the luteolin effects on ceramide metabolism, by increasing ceramide and reducing complex sphingolipids (Fig 4B).

### Luteolin unbalances the sphingolipid rheostat by inhibiting SphK2

**Fig 5:**
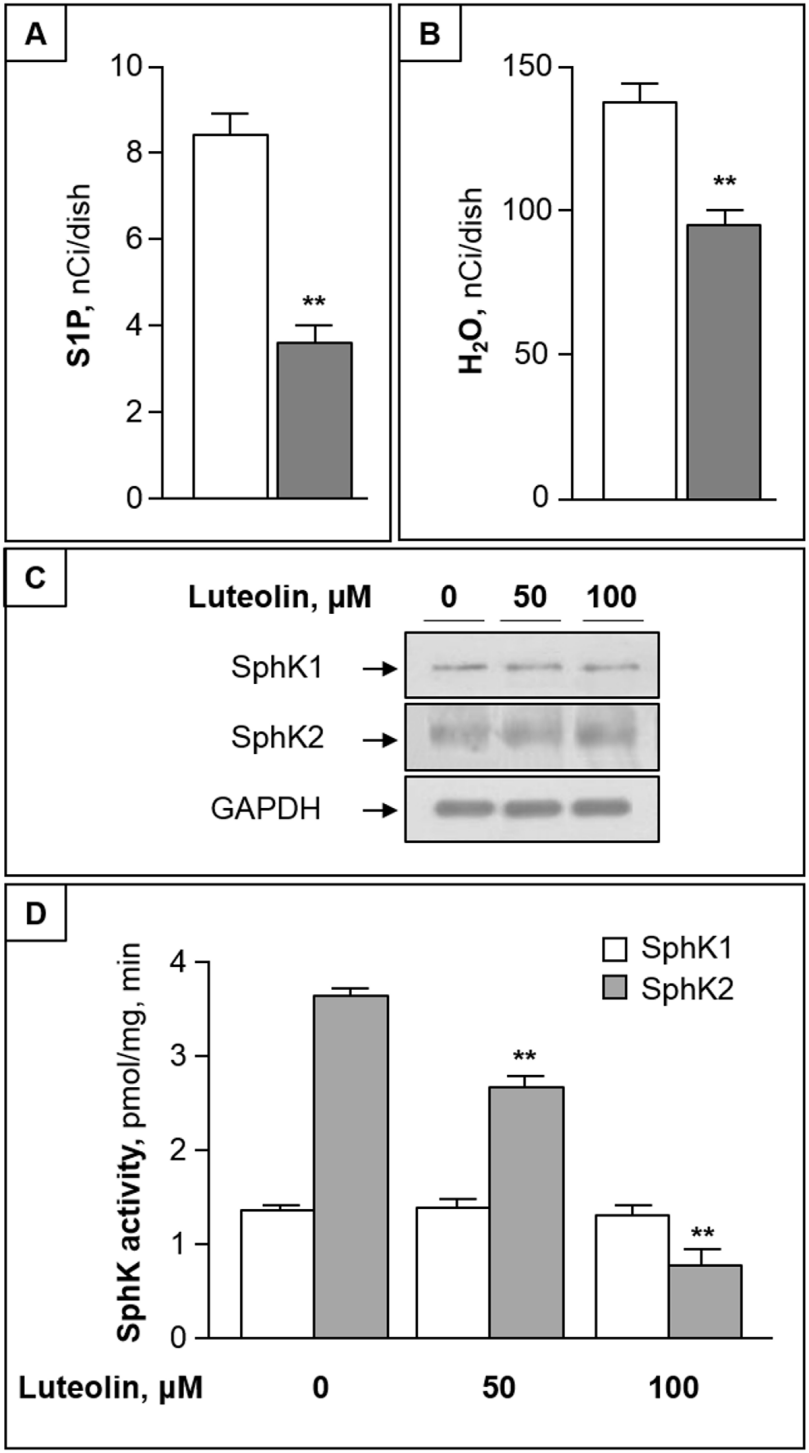
Luteolin reduces cellular S1P by inhibiting SphK2. (A) and (B) CC cells were pulsed with ^3^H-Sph for 2 h in the absence (white bar) or presence of 100 μM luteolin (grey bar). At the end, labeled S1P (A) and water (B) were evaluated in cells and medium, respectively. (C) CC cells were treated with 50–100 μM luteolin for 2h, and equal amounts of proteins were then analyzed for SphK1 and SphK2 proteins by immunoblotting. GAPDH was used as loading control. (D) SphK1 and SphK2 activities were assayed in the absence or presence of luteolin, using CC cell homogenate as enzyme source. Data are mean ± S.D. of three experiments in duplicate. **, p < 0.01 vs. control.

The analyses of the 1-phosphorylation metabolites of ^3^H-Sph (including ^3^H-S1P and ^3^H-water, its degradation product) revealed that both S1P- and water-associated radioactivity were significantly reduced by luteolin (Fig 5A and 5B). The finding that luteolin induced a decrease of both S1P and its degradation product prompted us to evaluate the possible effect of cytotoxic concentrations of luteolin on SphK expression and activity. Western blots revealed no appreciable differences in the expression of both SphK1 and SphK2 proteins (Fig 5C). Of interest, we found that 50–100 μM luteolin significantly inhibited SphK2 activity in a dose dependent fashion, but were ineffective on SphK1 (Fig 5D).

The luteolin-induced inhibition of both ceramide anabolism and SphK2 activity led to a significant increase of the ceramide/S1P ratio (more than 6-fold, p < 0.01), that is a relevant unbalance of the sphingolipid rheostat.

### S1P reduction is functional to luteolin toxicity

**Fig 6:**
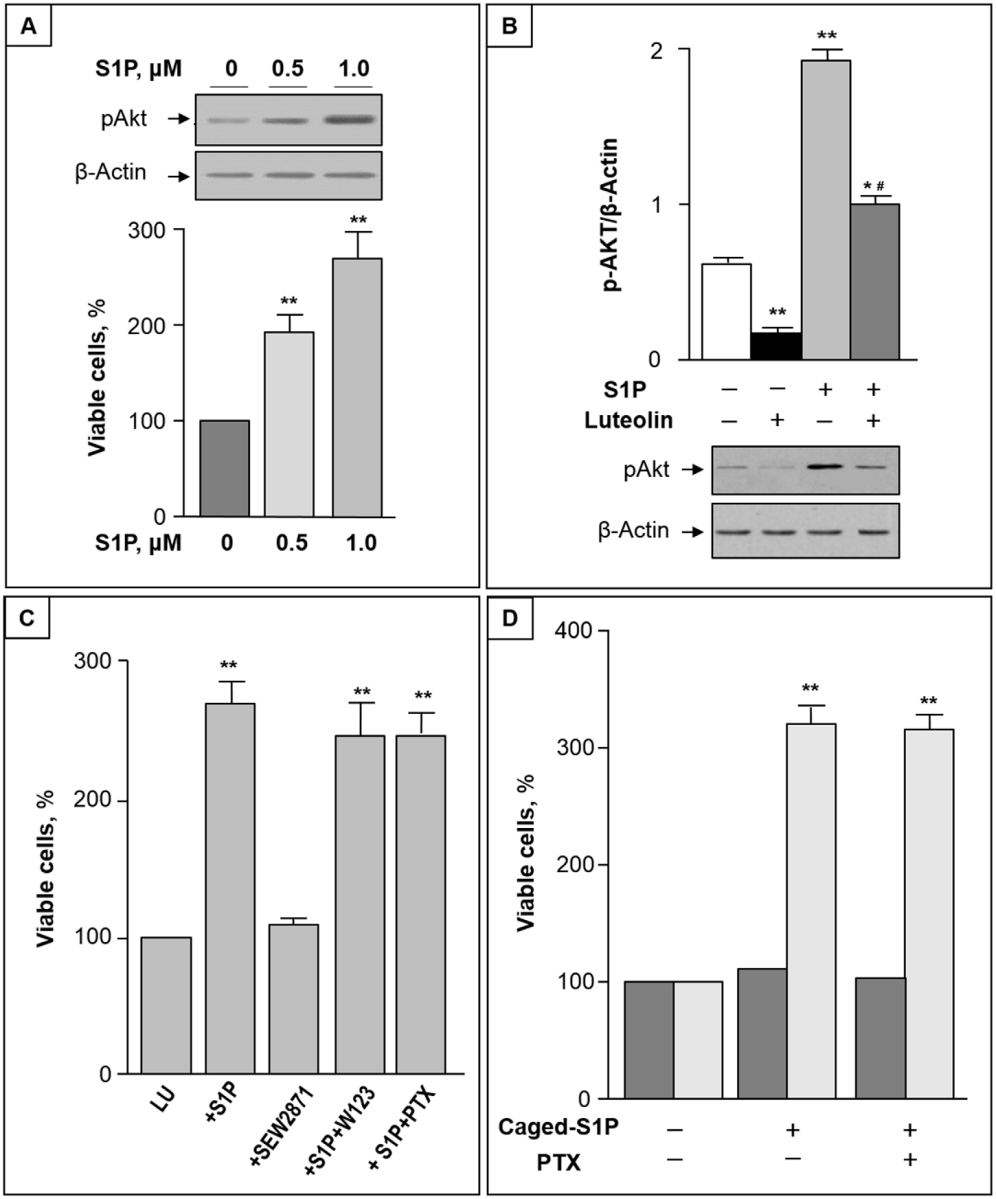
S1P protects CC cells from luteolin-induced toxicity by activating Akt. (A) Upper panel: CC cells were treated with 0.5–1 μM S1P for 2 h. Equal amounts of cell proteins were then analyzed for phosphorylated Akt (pAkt) by immunoblotting. Lower panel: CC cells were treated with different concentrations of S1P in the presence of 50 μM luteolin, and after 48 h cell viability was analyzed by MTT assay. (B) P-Akt/β-actin ratio (mean ± S.D) prior and after treatment of CC cells with 1 μM S1P and/or 50 μM luteolin for 2 h. *, p < 0.05 and **, p < 0.01 vs. untreated cells; #, p < 0.05 vs. luteolin-treated cells. A representative Western blot is reported in the lower part. (C) CC cells were incubated with luteolin alone (Lu) or in the presence of 1 μM S1P and/or 1 μM SEW2871; 5 μM W123; or 100 ng/ml PTX. (D) CC cells were incubated with luteolin in the absence or presence of 1 μM caged S1P without or with 100 ng/ml PTX for 48 hours. Cell viability was measured without (dark grey) or with (light grey) UV irradiation for 30 s. In (C) and (D), cell viability was determined by MTT assay, and the viability of luteolin-treated cells was regarded as 100%. Data are mean ± S.D. of at least two independent experiments. **, p < 0.01.

The luteolin-induced reduction of intracellular S1P led us to evaluate whether S1P administration affects luteolin cytotoxicity. We first found that S1P treatment significantly increased Akt phosphorylation in CC cells (Fig 6A, up). In addition, co-administration of S1P and luteolin resulted in a significant increase of viable cells (Fig 6A, down), and reduced the luteolin inhibition of Akt phosphorylation (Fig 6B).

To determine whether S1P exerts protective effects on luteolin-induced death through a pathway that involves S1PRs, two pharmacological inhibitors with different mechanisms of action were utilized. Since binding of S1P to S1P1 receptor has been shown to be involved in colitis-induced cancer [33], we first evaluated the possible role of S1P1 receptor in S1P effect on CC cells. The specific S1P1 agonist SEW2871 was unable to mimic S1P protective-effect on luteolin toxicity (Fig 6C), and the S1P antagonist W123 was without relevant effects on S1P-mediated survival of CC cells (Fig 6C, lanes 3 and 4, respectively). To address the question whether S1P induced pro-survival effects were dependent of its specific G-protein coupled receptors, we assayed cell survival in the presence of PTX, because all known S1P receptors are, at least in part, coupled with Gi/o protein [34]. As shown in Fig 6C, PTX was unable to influence the stimulatory effect of S1P on cell survival, suggesting that PTX-sensitive G-proteins are not involved in the signaling pathways of S1P enhancement of cell survival. Finally, to investigate the ability of S1P to act as intracellular mediator, we loaded CC cells with a photolysable (caged) derivative of S1P. As shown in Fig 6D, after photolysis, caged S1P exhibited a significative, protective effect against luteolin-induced cell death, and this effect remained unmodified.in the presence of PTX.

## Discussion

In this study, we initially report that, luteolin displays a dose-dependent apoptotic effect on CC cells in the range of 50–200 μM, known as physiological concentration of dietary polyphenols in the gastro-intestinal tract [35]. Of relevance, in the same range of concentrations, the flavone showed no toxicity in DEs, used as model of normal intestinal epithelial cells. Thus it emerges that luteolin exhibit cytotoxic activity toward human CC cells with little or no effect on normal cells, suggesting it might represent an ideal candidate for new therapeutics.

Our study also reveals for the first time that an increased content of cellular ceramide is at the helm of the different sensitivity of DEs and CC cells to luteolin toxicity. We initially found that, in the used culture conditions, CC cells showed approximately half the levels of ceramide when compared with DEs. Of interest, a similar decrease in the cellular content of ceramide was found in human colon cancer when compared with normal colon mucosa (15), suggesting that CC cells are particularly sensitive to the elevation of ceramide. In addition, we found that luteolin treatment enhanced ceramide level in CC cells, but not in DEs. Pulse experiments with labeled Sph and serine revealed that both the recycling pathway, and *de novo* pathway of ceramide synthesis are involved in the luteolin-induced increase of ceramide in CC. Since both pathways involve ceramide synthase proteins, that show differential specificities in regard to acyl chain length [36], the luteolin-induced regulation of a specific pool of ceramide, with restricted acyl chain lengths, and possibly apoptotic properties, cannot be excluded.

Noteworthy, the ceramide elevation in CC cells occurred rapidly, and prior to their death, suggesting a role for ceramide as a mediator of luteolin toxicity. In agreement, an induced elevation of ceramide content in CC cells led to their apoptotic death, supporting the hypothesis that ceramide generated through the breakdown of dietary sphingolipids, may protect against intestinal tumorigenesis [17].

Newly synthesized ceramide formed in the ER needs to reach the Golgi apparatus for the biosynthesis of complex sphingolipids; thus, a crucial step in its anabolic processing is represented by its ER-Golgi transport. Besides the protein(CERT)-mediated transport of ceramide that acts mainly for sphingomyelin biosynthesis [37], neo-synthesized ceramide in the ER can move to Golgi through a vesicle-mediated route, and this transport is functional to both sphingomyelin and glucosylceramide biosynthesis [38]. Two different experimental approaches led us to demonstrate that luteolin impaired the ER/Golgi transport of ceramide, and thus to reveal that the alteration of this traffic is involved in the increased ceramide/sphingolipid ratio of CC cells upon luteolin treatment. First, using BODIPY-C_5_-Cer, a fluorescent ceramide derivative known to mimic the ER-Golgi trafficking of the natural counterpart [30], the Golgi localization of ceramide was found altered by luteolin treatment. Then, the use of BFA, which fuses Golgi membranes to the ER [39], thus rendering ceramide directly available for complex sphingolipid biosynthetic enzymes, revealed that CC cells were no more sensitive to luteolin-induced ceramide accumulation, as well as to sphingomyelin and glucosylceramide decrease. Overall, these results demonstrate a ceramide translocation defect as a must for luteolin effects on sphingolipid metabolism.

Among different protein kinases, PI3K/Akt has emerged in the regulation of the ER-Golgi traffic [40], and we recently reported that the ceramide transport from ER to Golgi is controlled by phosphorylated Akt in some extra colonic cell lines [41, 42]. Our data demonstrate that luteolin effectively inhibits Akt phosphorylation in CC cells, and that the Akt inhibitor LY294002 was able to mimic luteolin in inhibiting ceramide metabolism to complex sphingolipids. Thus, the inhibition of Akt phosphorylation emerges as a key mechanism affecting the ER-Golgi transport of ceramide in the used colon cancer cell model, and involved in CC cells apoptosis. The discrepancy of luteolin toxicity observed in CC cells and DEs may be explained by the difference in Akt phosphorylation. In an immune-histochemical study of colonic tissue, the expression of phosphorylated Akt was detectable in CC specimens but not in normal colonic epithelium [43], suggesting Akt phosphorylation is essential for survival of colon cancer cells but not normal enterocytes.

Of relevance, luteolin not only increased the levels of pro-apoptotic ceramide, but also inhibited the production of its antagonist S1P. S1P is produced intracellularly by two closely related SphKs, named SphK1 and SphK2, which are crucial in the modulation of the sphingolipid rheostat [11, 44]. Our results demonstrate for the first time that luteolin can act as inhibitor of SphK2, the predominant SphK isoform in the used CC cell line, as well as in several colon cancer cell lines [45], but exerts only a modest, if any, effect on SphK1 activity. Experiments are ongoing in our laboratory to clarify the mechanisms underlying luteolin inhibition of SphK2.

Both SphK1 and SphK2 have been widely implicated in carcinogenesis, with high expression levels correlated with poor patient survival [46, 47]. Different studies reported that SphK1 is overexpressed in human colon cancer and in a murine model of colon carcinomas [19, 48], and promotes malignant progression of this disease [33, 49]. In spite the role of SphK2 in colon cancer remains unclear, this isoform was shown to contribute to oxaliplatin resistance in human colon cancer cells [45], and its inhibition by sodium butyrate results in colon cancer cell apoptosis [50]. We found that S1P is able to reduce the cytotoxic effects of luteolin, supporting that the inhibition of SphK2, and the consequent reduction of S1P contribute to regulate ceramide levels and luteolin toxicity in CC cells. Indeed, after administration of S1P, CC cells exhibited a significant reduced sensitivity to the cytotoxic effect of luteolin.

S1P exerts signaling roles through acting both as a ligand for a family of S1P-specific receptors, at low nM concentrations, and, at higher concentrations, as a modulator of a range of intracellular proteins [11, 46]. Previous studies reported that S1P1 receptor is expressed in human colon cancer cells [51], and is involved in colitis-induced cancer [33]. However, the present results obtained with S1P1 agonist/antagonist and PTX demonstrate that S1P did not signal either via S1P1 or G protein-coupled receptors to induce CC cell survival, and suggest it acts via intracellular mechanisms. The findings that luteolin reduces the intracellular levels of S1P, and that 0.5–1 μM S1P (that is 10–100 fold higher that the Kd for S1P binding to its receptors [52]) was effective in protecting CC cells from luteolin toxicity appear consistent with an intracellular action of S1P. To confirm this hypothesis, we used a previously characterized photolysable S1P derivative to release S1P inside CC cells [53]. Here we show that caged S1P exerts a pro-survival effect on luteolin-induced toxicity, and this effect was maintained in the presence of PTX, ruling out the possibility that S1P generated from photolysis of intracellular caged S1P might leech out and activate G protein-coupled receptor. Therefore our results strongly suggest that intracellular S1P can activate Akt to promote CC cell survival. In support, in different cells, it was reported that intracellular S1P is critical for Akt activation and promotion of cell survival, independently of S1P receptors [54–56].

Overall, our study demonstrates for the first time that luteolin exhibits pro-apoptotic activities in CC cells not only by its inhibitory effect on Akt phosphorylation, but also by a direct inhibition of SphK2, with consequent reduction of the S1P-mediated phospho-Akt stimulation. As consequences, first Akt phosphorylation is deregulated, then the ER-Golgi traffic of ceramide is impaired, and finally the sphingolipid rheostat is unbalanced. Thus, the results presented here shed new light on the mechanisms underlying luteolin effects, implicating this flavone as a natural molecule able to unbalance the sphingolipid rheostat by tipping it to the side of death. Targeting the sphingolipid rheostat with a diet enriched/supplemented with luteolin emerges as a potential strategy to improve existing treatments in CRC, and future investigations of this strategy are promoted.

Our study deserves a final comment. Increasing evidence demonstrates that targeting SphKs has a promising potential as an anti-cancer strategy [46, 47], and this has boosted the field of SphKs inhibitor research, leading to the synthesis of an impressive number of potential molecules to target SphKs [57–59]. The finding that the natural phytomolecule luteolin can act as a SphK2 inhibitor may have implications in this field, and in developing this class of inhibitors.
